# Lower regional urbanicity and socioeconomic status attenuate associations of green spaces with hypertension and diabetes mellitus: a national representative cross-sectional study in China

**DOI:** 10.1265/ehpm.24-00121

**Published:** 2024-09-07

**Authors:** Wanzhou Wang, Chao Yang, Jinwei Wang, Fulin Wang, Ze Liang, Yueyao Wang, Feifei Zhang, Chenyu Liang, Chenshuang Li, Yiqun Lan, Shuangcheng Li, Pengfei Li, Ying Zhou, Luxia Zhang, Lieyun Ding

**Affiliations:** 1 National Institute of Health Data Science at Peking University, Beijing 100191, China; 2 Institute of Medical Technology, Peking University Health Science Center, Beijing 100191, China; 3 Renal Division, Department of Medicine, Peking University First Hospital, Peking University Institute of Nephrology, Beijing 100034, China; 4 Research Units of Diagnosis and Treatment of Immune-Mediated Kidney Diseases, Chinese Academy of Medical Sciences, Beijing 100034, China; 5 Advanced Institute of Information Technology, Peking University, Hangzhou 311215, China; 6 Key Laboratory of Chronic Kidney Disease Prevention and Treatment, Peking University, Ministry of Education of the People’s Republic of China, Beijing, China; 7 Key Laboratory for Earth Surface Processes of the Ministry of Education, College of Urban and Environmental Sciences, Peking University, Beijing 100871, China; 8 Center for Smart and Healthy Buildings, Huazhong University of Science and Technology, Wuhan, Hubei 430074, China

**Keywords:** Urbanization, Regional urbanicity, Residential greenness, Socio-economic status, High blood pressure, Diabetes mellitus

## Abstract

**Background:**

High blood pressure (HBP) and diabetes mellitus (DM) are two of the most prevalent cardiometabolic disorders globally, especially among individuals with lower socio-economic status (SES). Studies have linked residential greenness to decreased risks of HBP and DM. However, there has been limited evidence on whether SES may modify the associations of residential greenness with HBP and DM.

**Methods:**

Based on a national representative cross-sectional study among 44,876 adults, we generated the normalized difference vegetation index (NDVI) at 1 km spatial resolution to characterize individuals’ residential greenness level. Administrative classification (urban/rural), nighttime light index (NLI), individual income, and educational levels were used to characterize regional urbanicity and individual SES levels.

**Results:**

We observed weaker inverse associations of NDVI with HBP and DM in rural regions compared to urban regions. For instance, along with per interquartile range (IQR, 0.26) increment in residential NDVI at 0∼5 year moving averages, the ORs of HBP were 1.04 (95%CI: 0.94, 1.15) in rural regions and 0.85 (95%CI: 0.79, 0.93) in urban regions (P = 0.003). Along with the decrease in NLI levels, there were continuously decreasing inverse associations of NDVI with DM prevalence (P for interaction <0.001). In addition, weaker inverse associations of residential NDVI with HBP and DM prevalence were found among individuals with lower income and lower education levels compared to their counterparts.

**Conclusions:**

Lower regional urbanicity and individual SES could attenuate the associations of residential greenness with odds of HBP and DM prevalence.

**Supplementary information:**

The online version contains supplementary material available at https://doi.org/10.1265/ehpm.24-00121.

## Graphical Abstract

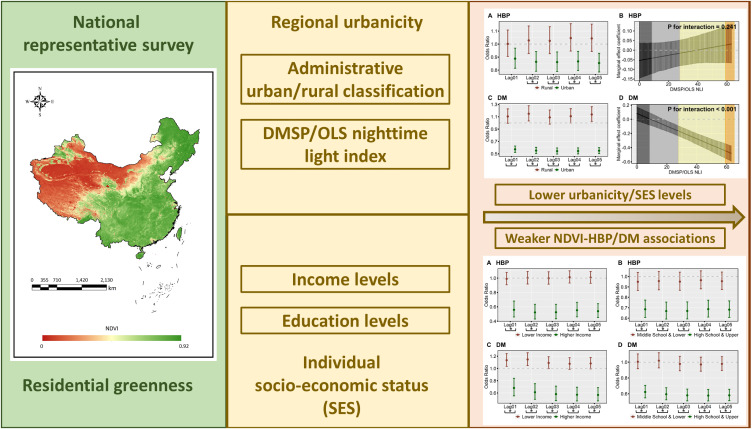

## 1. Introduction

Hypertension (high blood pressure, HBP) and diabetes mellitus (DM) are two of the most common cardiometabolic disorders worldwide [1, 2]. According to the statistics from the Global Burden of Disease (GBD) survey, the global prevalence of high systolic blood pressure (SBP) among adults increased from 2.18 billion in 1990 to 4.06 billion in 2019, contributing to 154 million and 235 million disability-adjusted life years (DALYs) in the years 1990 and 2019, respectively [1, 3]. Meanwhile, a total of 134 million deaths can be attributable to high fasting plasma glucose globally between 1990 and 2019, with 2.91 million in 1990 rising to 6.50 million deaths in 2019 [3]. Notably, a growing body of global evidence has linked regional urbanicity and individual SES to HBP and DM [4–6]. A study among 1,211,386 participants from 76 low- and middle-income countries (LMICs) indicated that HBP may increasingly affect individuals in rural regions and those with lower SES [5]. A prospective cohort study among 53,891 individuals in China observed increased risks of incident HBP among individuals with lower SES compared to those with higher SES [7]. As for DM, a prospective cohort study among over 1.9 million employees in Denmark also identified elevated risks of DM among participants with lower SES [8].

Under the global context of urbanization, the beneficial health effects of green spaces have been increasingly reported [9]. Studies across countries have investigated the associations between residential greenness and HBP [9–17] and DM [10, 18–26]. Generally, the findings were still insufficient and inconclusive given the geographic and demographic disparities across the studies [18, 27]. Further empirical evidence is warranted for health promotion through green space planning, particularly investigations based on large population and geographic scales from underdeveloped regions [9].

Empirical studies investigating the health effects of green spaces should consider the heterogeneity of regional settings associated with the process of urbanization [22, 28]. Urbanization can contribute to the development of regional healthcare settings and accessibility to healthcare services, which may amplify the beneficial health effects of green spaces [29]. However, the emissions of ambient air pollution and unhealthy lifestyle behaviors associated with urbanization may also reduce the health benefits [29]. In addition, individual SES also influences the utilization of green spaces [30–32], and may thus potentially modify the health effects.

Nevertheless, the effect modification by regional urbanicity and individual SES levels on associations of residential greenness with HBP and DM has been insufficiently investigated to date. Most previous studies have been limited in geographic distribution, or conducted solely in either urban or rural regions [11, 33–38]. It is still inconclusive whether the beneficial health effects of residential greenness can vary across regional urbanicity settings or individual SES levels [12].

Developing regions such as China are confronting significant inequality in regional urbanicity, individual SES development, and green space planning [39, 40]. Elucidating the complex interplay among regional urbanicity, individual SES, and residential greenness can facilitate evidence-based green space planning aimed at promoting population health and well-being. To this end, based on individual survey records and high-solution remote sensing products, this national representative cross-sectional study among 44,876 adults investigated the associations of residential greenness with HBP and DM, as well as the effect modification by regional urbanicity and individual SES on the associations.

## 2. Methods

### 2.1 Study participants and sampling methods

This national representative cross-sectional study, the China National Survey of Chronic Kidney Disease (CNSCKD) adopted a multistage stratified sampling process. The study was carried out between the years 2007 and 2010, covering over 47,000 urban and rural residents from 13 provinces/autonomous regions/municipalities in mainland China. First, the provinces were chosen using probability-proportional-to-size (PPS) sampling. Second, 1 urban and 1 rural district of each province were selected using simple random sampling. Third, 3 sub-districts were selected from each district using simple random sampling. Fourth, 5 communities from each sub-district were further randomly sampled. In total, 47,204 participants aged ≥18 years completed the study. The detailed sampling procedure is shown in Fig. S1.

Demographic characteristics, lifestyle behaviors, and disease-related information were collected by standardized questionnaires and laboratory examinations by trained investigators. More details have been reported previously [41]. Finally, a total of 44,876 participants with completed questionnaires and health examinations were included in the final analysis.

Before the beginning of the study, all the participants signed written informed consents. The protocol of this study has been approved by the Biomedical Ethics Review Committee of Peking University (Approval number: IRB00001052-20030).

### 2.2 Residential greenness

This study used the normalized difference vegetation index (NDVI) to characterize residential greenness levels based on the geocoded residential address of each participant [42]. Considering a high reflectance in the near-infrared radiation (NIR) channel and a low reflectance in the red reflectance (Red) channel of plants, the NDVI is a normalized transform of the NIR to Red ratio calculated using the formula:

$$\text{NDVI}=\frac{\mathrm{NIR}-\mathrm{Red}}{\mathrm{NIR}+\mathrm{Red}}$$

Previous studies reported significant associations between NDVI and green coverage (with the pseudo-R-squared of 0.77–0.80) [43]. In this study, we acquired SPOT VEGETATION NDVI data with geometric and atmospheric corrections from the year 2002 to 2010 with a spatial resolution of 1 km * 1 km and a temporal resolution of 10 days in China (Resource and Environment Data Cloud Platform, http://www.resdc.cn/) [44, 45]. The NDVI data have been widely used to characterize greenness in epidemiological studies in China [9, 42]. More detailed information is provided in the supplementary materials.

### 2.3 Regional urbanicity and individual SES levels

Two methods were used to evaluate the regional urbanicity level [46, 47]. First, the National Bureau of Statistics in China was adopted to classify administrative urban/rural regions. Second, we used high-resolution nighttime light index (NLI) time-series data derived from the Defense Meteorological Satellite Program (DMSP)/Operational Line-scan System (OLS) (http://ngdc.noaa.gov/eog/dmsp/downloadV4composites.html) [47]. The DMSP/OLS NLI data can sensitively and accurately estimate regional gross domestic product and socio-economic development and have been widely used as an indicator to represent regional urbanicity level [46, 48, 49]. More detailed information is provided in the supplementary materials.

Individual SES levels, including income and education levels, were characterized using questionnaires in the CNSCKD survey. Income was categorized into 3 levels (<500; 500∼1000; and >1000 yuan per capita per month). Education levels were categorized into 5 levels (primary school and lower; primary school; middle school; high school; and college and upper).

### 2.4 Outcomes

Hypertension was defined when (a) resting SBP ≥140 mm Hg or resting diastolic blood pressure (DBP) ≥90 mm Hg, or (b) having a self-reported history of physician-diagnosed HBP, or (c) using anti-hypertensive medication in the past 14 days. DM was defined when (a) fasting plasma glucose (FPG) ≥7.0 mmol/L, or (b) having a self-reported history of physician-diagnosed DM, or (c) taking hypoglycemic agents. The resting blood pressure measurements and laboratory examinations were carried out by technicians with medical training following standardized procedures [41].

### 2.5 Confounders

This study collected information on several demographic and lifestyle behavior variables that could potentially confound the associations of residential greenness with HBP and DM through a questionnaire [10, 12, 50, 51]. The covariates included sex (categorized by female/male), age, body mass index (BMI), smoking status (categorized by non-smoking/current smoking), alcohol drinking (categorized by <1, 1∼2, and ≥3 times per week), physical activity level (the total time of various types of physical activities including dancing, running, and others, during leisure time in the last month, categorized by <3 and ≥3 hours/week), dietary vegetable intake (categorized by <250, 250∼500, and ≥500 g/day), and season (categorized into cold season, November∼March, and warm season, April∼October). In addition, the DMSP/OLS NLI level of each participant’s residential address was also included as a confounder.

### 2.6 Statistical analyses

The moving averages of NDVI exposure data from the investigation year (Lag0) to 1∼5 years (Lag01∼Lag05) preceding the investigation year of each participant were generated. For instance, Lag03 represented the moving averages of the NDVI at Lag0, Lag1 (the year before the investigation year), Lag2, and Lag3. The DMSP/OLS data at the same exposure metrics were also generated and assigned to each participant.

We used the generalized additive model (GAM) to quantitively evaluate the associations of residential NDVI with HBP and DM after adjusting the confounders. To assess the effect modification by regional urbanicity and individual SES on the associations of NDVI with HBP and DM, we also conducted stratified analyses based on administrative classification (urban and rural), income (lower income, ≤1000 *yuan* per capita month, and higher income, >1000 *yuan* per capita month), and education level (lower education, middle school and lower, and higher education, high school and upper). The subgroup differences were examined using the *Z*-statistics [52]. The results were shown as estimated odds ratio (OR) of HBP and DM associated with per interquartile range (IQR, 0.26) increase in NDVI. Meanwhile, we included the multiplicative interaction term of DMSP/OLS NLI and NDVI (including their main effect terms) in the model and calculated the conditional effects of NDVI on the predicted probability of HBP and DM across regions with different NLI levels [53].

Several sensitivity analyses were further conducted to evaluate the robustness of the above results. First, to characterize the potential non-linear associations of NDVI with HBP and DM, we used a cubic spline function (k was set to be 3) instead of the linear term in the model. Second, to exclude the potential confounding effects of other major environmental factors, we also included ambient fine particulate matter (PM_2.5_), ozone (O_3_) pollution, or temperature in the model [46, 54–56]. Detailed information on related remote-sensing products is provided in the supplementary materials. Third, we conducted multicollinearity assessments for all the statistical models using variance inflation factor (VIF) analysis, and a tolerance value of 0.20 (VIF = 5) was set to be the threshold [57].

A two-sided P < 0.05 was defined as statistical significance. The statistical analyses were carried out using the R software (Version 4.3.0, https://cran.r-project.org/).

## 3. Results

### 3.1 Characteristics of the study participants

As shown in Table 1 & Table S1, after excluding missing values, a total of 44629 participants (15328 HBP and 29301 non-HBP participants) and 44833 participants (10521 DM and 34312 non-DM) were included in the final analyses, respectively. Compared to non-HBP participants (50.9%), a higher proportion (53.0%) of HBP participants were from urban regions (P < 0.001). Meanwhile, compared to HBP participants, non-HBP participants were generally with larger proportions of higher income (>1000 *yuan* per capita month, 21.4% vs 18.9%) and higher education levels (college and upper, 21.1% vs 11.7%; high school, 26.5% vs 22.1%) (P < 0.001). Similar patterns were also found among the DM and non-DM participants.

**Table 1:** Baseline characteristics of the participants from the CNSCKD survey, stratified by HBP or DM.

**Characteristics**	**HBP** **(N = 15328)**	**Non-HBP** **(N = 29301)**	**P-value**	**DM** **(N = 10521)**	**Non-DM** **(N = 34312)**	**P-value**
Region			<0.001			<0.001
Rural	7209 (47.0%)	14387 (49.1%)		4805 (45.7%)	16909 (49.3%)	
Urban	8119 (53.0%)	14914 (50.9%)		5716 (54.3%)	17403 (50.7%)	
Income (*yuan*, per capita month)			<0.001			<0.001
≤500	5736 (37.4%)	10189 (34.8%)		3814 (36.3%)	12158 (35.4%)	
500∼1000	4240 (27.7%)	6947 (23.7%)		2690 (25.6%)	8537 (24.9%)	
>1000	2892 (18.9%)	6279 (21.4%)		1789 (17.0%)	7405 (21.6%)	
Not reported	2460 (16.0%)	5886 (20.1%)		2228 (21.2%)	6212 (18.1%)	
Education			<0.001			<0.001
Below primary school	2410 (15.7%)	2209 (7.5%)		1367 (13.0%)	3253 (9.5%)	
Primary school	3558 (23.2%)	4796 (16.4%)		2395 (22.8%)	5977 (17.4%)	
Middle school	4137 (27.0%)	8284 (28.3%)		2951 (28.0%)	9502 (27.7%)	
High school	3384 (22.1%)	7762 (26.5%)		2341 (22.3%)	8855 (25.8%)	
College and upper	1799 (11.7%)	6178 (21.1%)		1440 (13.7%)	6633 (19.3%)	
Not reported	40 (0.3%)	72 (0.2%)		27 (0.3%)	92 (0.3%)	
Sex			<0.001			<0.001
Male	7121 (46.5%)	12009 (41.0%)		4680 (44.5%)	14540 (42.4%)	
Female	8207 (53.5%)	17292 (59.0%)		5841 (55.5%)	19772 (57.6%)	
Age (years)	57.4 ± 13.9	45.5 ± 14.3	<0.001	56.2 ± 14.0	47.5 ± 15.0	<0.001
BMI (kg/m^2^)	25.4 ± 3.7	23.0 ± 3.4	<0.001	25.0 ± 3.7	23.5 ± 3.6	<0.001
Smoking status			<0.001			0.724
Non-smoking	11545 (75.3%)	22592 (77.1%)		8042 (76.4%)	26251 (76.5%)	
Current smoking	3772 (24.6%)	6685 (22.8%)		2468 (23.5%)	8034 (23.4%)	
Not reported	11 (0.1%)	24 (0.1%)		11 (0.1%)	27 (0.1%)	
Alcohol drinking			<0.001			<0.001
<1 time a week	12556 (81.9%)	25102 (85.7%)		8652 (82.2%)	29178 (85.0%)	
1∼2 times a week	815 (5.3%)	1612 (5.5%)		516 (4.9%)	1925 (5.6%)	
>3 times a week	1932 (12.6%)	2521 (8.6%)		1332 (12.7%)	3136 (9.1%)	
Not reported	25 (0.2%)	66 (0.2%)		21 (0.2%)	73 (0.2%)	
Physical activity level			<0.001			<0.001
<3 hours/week	8467 (55.2%)	16339 (55.8%)		5418 (51.5%)	19461 (56.7%)	
≥3 hours/week	4134 (27.0%)	6511 (22.2%)		2624 (24.9%)	8059 (23.5%)	
Not reported	2727 (17.8%)	6451 (22.0%)		2479 (23.6%)	6792 (19.8%)	
Dietary vegetable intake			<0.001			<0.001
≥500 g/day	5911 (38.6%)	10425 (35.6%)		4397 (41.8%)	12025 (35.0%)	
250–500 g/day	6595 (43.0%)	12669 (43.2%)		4244 (40.3%)	15097 (44.0%)	
<250 g/day	2154 (14.1%)	4222 (14.4%)		1298 (12.3%)	5110 (14.9%)	
Not reported	668 (4.4%)	1985 (6.8%)		582 (5.5%)	2080 (6.1%)	

Note: Distributions are showed as number (percentage) for categorical variables or Mean ± Standard Deviation (SD) for continuous variables. Chi-square test and Student-t test were used to examine the subgroup differences.

As for other demographic characteristics, compared to non-HBP/non-DM participants, HBP/DM participants were associated with higher male proportion, age, BMI, and more frequent alcohol drinking (P < 0.001).

### 3.2 Associations of residential greenness with prevalence of HBP and DM among the general population

**Fig. 1:**
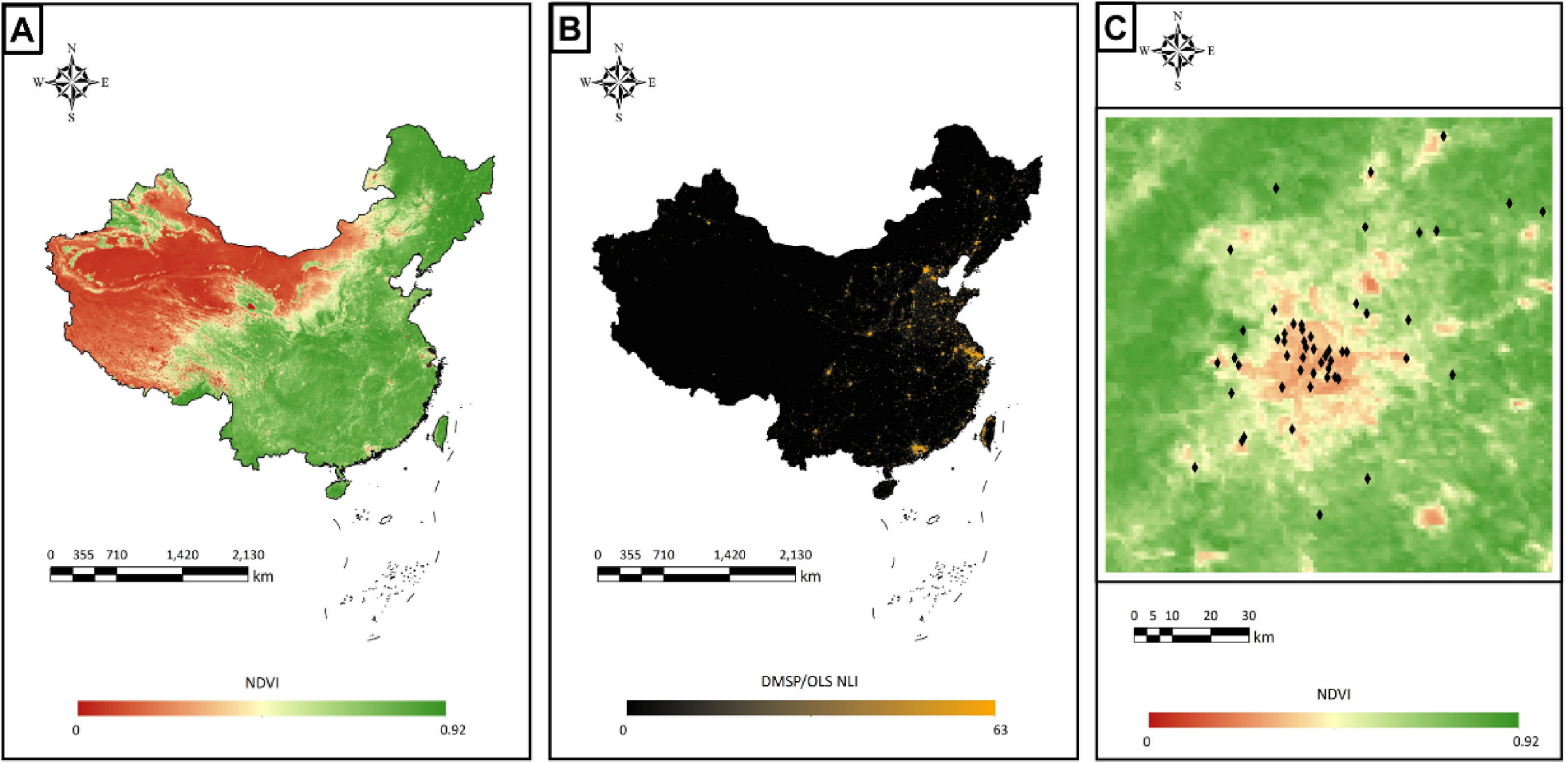
Distributions of NDVI and NLI levels in 2010.

Distributions of NDVI and NLI levels in 2010 are shown in Fig. 1. The Spearman correlation coefficients between NDVI at different exposure metrics ranged from 0.986∼0.997. Meanwhile, the Spearman correlation coefficients between NDVI and NLI ranged from −0.734 to −0.781, indicating strong negative correlations (Table S2).

**Fig. 2:**
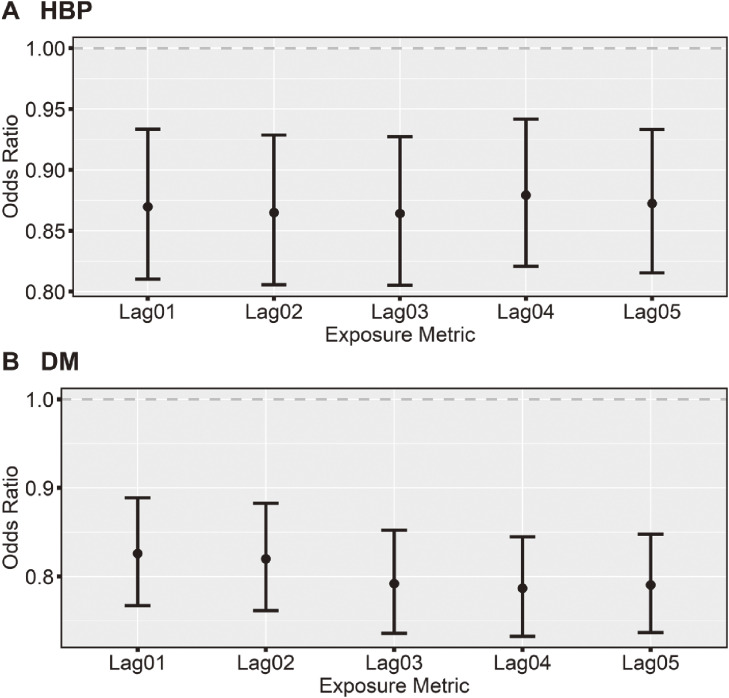
Associations of residential NDVI with HBP and DM among the general population.

The associations of residential greenness with the prevalence of HBP and DM among the general population are shown in Fig. 2 (the effect estimates are shown in Table S3). We found residential greenness was associated with decreased odds of HBP and DM among the general population. Specifically, per IQR (0.26) increment in residential NDVI value was associated with ORs of 0.86 (95%CI: 0.81, 0.93, Lag03) for HBP and 0.79 (95%CI: 0.73, 0.84, Lag04) for DM.

### 3.3 Effect modification by regional urbanicity on associations of residential NDVI level with prevalence of HBP and DM

**Fig. 3:**
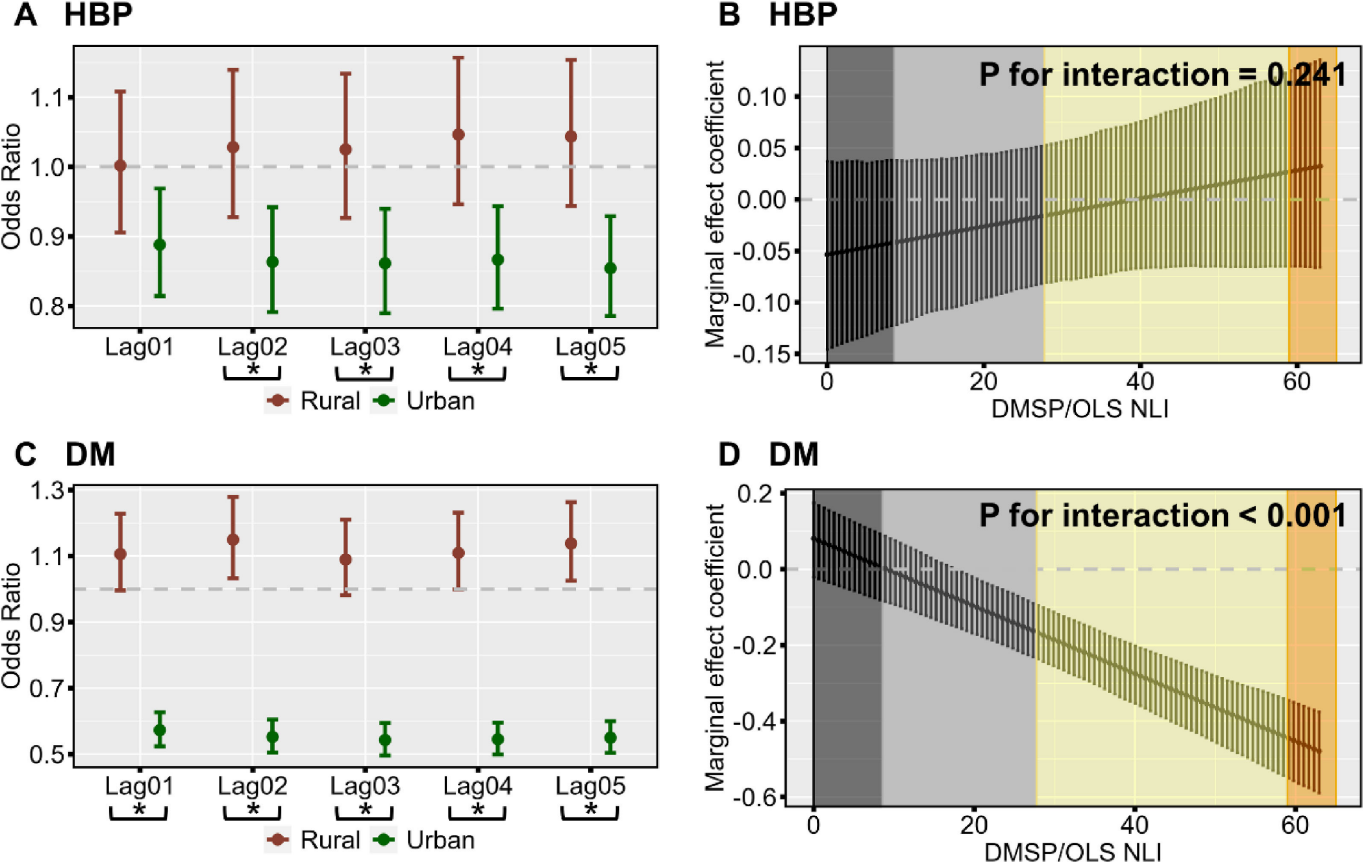
Effect modification by regional urbanicity on associations of residential NDVI with HBP and DM.

Figure 3(A, C) shows the effect modification by regional administrative urban/rural classification on associations of residential NDVI level with the prevalence of HBP and DM. Generally, we observed weaker inverse associations of NDVI with HBP and DM in rural regions compared to urban regions. For instance, along with per IQR increment in residential NDVI at Lag05, the ORs of HBP were 1.04 (95%CI: 0.94, 1.15) in rural regions and 0.85 (95%CI: 0.79, 0.93) in urban regions (P for subgroup difference = 0.003), and the ORs of DM were 1.14 (95%CI: 1.03, 1.26) in rural regions and 0.55 (95%CI: 0.50, 0.60) in urban regions (P for subgroup difference <0.001). The detailed effect estimates are shown in Table S4. The effect modification by regional DMSP/OLS NLI levels on associations of residential NDVI level with prevalence of HBP and DM is further shown in Fig. 3(B, D). The results indicated that along with the decrease in NLI levels, there were continuously decreasing inverse associations of residential NDVI with DM prevalence (P for interaction terms of NDVI and NLI <0.001). Nevertheless, no statistically significant effect modification by NLI on associations of NDVI with HBP was observed.

### 3.4 Effect modification by individual socio-economic status on associations of residential NDVI level with prevalence of HBP and DM

**Fig. 4:**
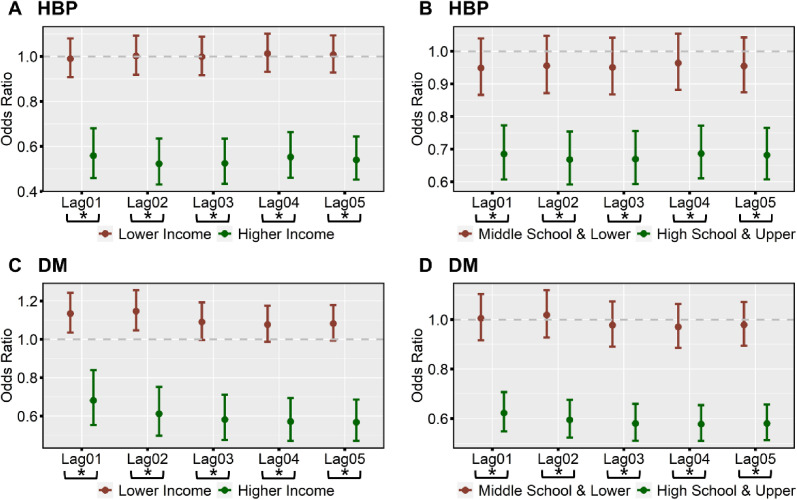
Effect modification by individual socio-economic status on associations of residential NDVI with HBP and DM.

The effect modification by individual income and education levels on associations of residential NDVI level with the prevalence of HBP and DM is shown in Fig. 4 & Table S5. In general, weaker inverse associations of residential NDVI with HBP and DM prevalence were found among individuals with lower SES levels. For instance, along with an IQR increment in residential NDVI at Lag05, the ORs were 1.01 (95%CI: 0.93, 1.09) and 0.54 (95%CI: 0.45, 0.64) for HBP (P for subgroup difference <0.001), and 1.08 (95%CI: 0.99, 1.18) and 0.57 (95%CI: 0.47, 0.69) for DM (P for subgroup difference <0.001) among individuals with lower and higher income, respectively. In addition, along with an IQR increment in residential NDVI at Lag05, the ORs were 0.95 (95%CI: 0.87, 1.04) and 0.68 (95%CI: 0.61, 0.77) for HBP (P for subgroup difference <0.001), and 0.98 (95%CI: 0.89, 1.07) and 0.58 (95%CI: 0.51, 0.66) for DM (P for subgroup difference <0.001) among individuals with lower and higher education levels.

### 3.5 Sensitivity analyses

**Fig. 5:**
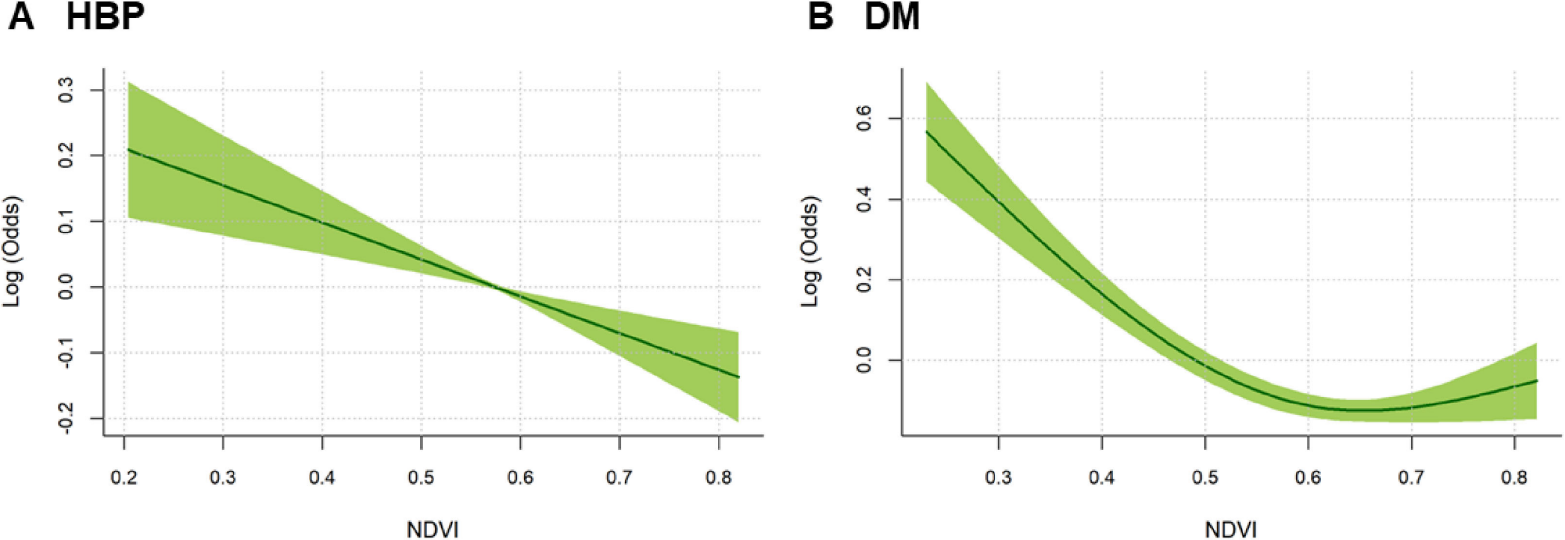
Exposure-response curves for associations of residential NDVI with HBP and DM.

The exposure-response curves for associations of NDVI with odds of HBP and DM prevalence were generally linear at all the exposure metrics (Lag01∼Lag05), as shown in Fig. 5 & Fig. S2. The effect modification by DMSP/OLS NLI on associations of NDVI with odds of HBP and DM prevalence showed similar patterns at all the exposure metrics, as shown in Fig. S3. These results further highlighted the robustness of our findings. Meanwhile, after further adjusting for the confounding effects of major air pollutants (PM_2.5_ and O_3_) and ambient temperature, the effect estimates remained robust (Table S6). The results indicated potential independent protective effects of residential greenness on HBP and DM. In addition, all the VIF values of the variables in our statistical models were <2.6, indicating no potential concerns of multicollinearity.

## 4. Discussions

HBP and DM are two of the most common prevalent cardiometabolic disorders leading to increasing disease burden worldwide. Elucidating the complex interactions between regional urbanicity, individual SES levels, and residential greenness can promote evidence-based green space planning and population health. Based on the nationwide CNSCKD survey data and high-solution NDVI and NLI products, this study investigated the associations of residential greenness with HBP and DM, and the effect modification by regional urbanicity and individual SES levels. We observed weaker inverse associations of NDVI with HBP and DM in rural regions and among individuals with lower income and education levels compared to their counterparts.

The beneficial effects of green spaces have been extensively reported under the global context of urbanization and urgent needs for built environment planning. The potential mechanisms supporting the beneficial effects of greenness on HBP and DM have also been reported by several studies. Studies have indicated that residential greenness can reduce risks of HBP and DM by eliminating hazardous environmental risk factors (e.g. ambient air pollutants and heat) with adverse effects on cardiometabolic health [9, 15, 58]. Recent studies across different geographic contexts have also linked residential greenness to microbiota diversity [59, 60], which may improve cardiometabolic health (e.g. blood pressure and FPG) by producing beneficial metabolites [61]. Meanwhile, greenness can reduce levels of stress hormones including glucocorticoids, adrenaline, and noradrenaline by reducing psychosocial stress and fostering social cohesion and interactions [15, 58, 62, 63]. These stress hormones have been linked to increased levels of blood pressure and FPG [64, 65]. In addition, greenness can regulate the activities of sympathetic/parasympathetic nerves and stabilize the autonomic nervous system [62, 66, 67], and may thus reduce the risks of HBP and DM [68].

Generally, previous studies investigating the associations of greenness with HBP and DM have yielded inconclusive findings [18, 27]. Our study observed that per IQR (0.26) increment in residential NDVI value was associated with ORs of 0.89 (95%CI: 0.84, 0.95) for HBP and 0.68 (95%CI: 0.64, 0.73) for DM. Our results were consistent with one cross-sectional study among urban dwellers from 33 communities in 3 cities in China, where a 0.17 unit increase in NDVI was associated with OR of 0.94 (95%CI: 0.91, 0.98) of HBP [34], and a 0.1 unit increase in NDVI was associated with OR of 0.88 (95%CI: 0.82, 0.94) of DM [50]. Meanwhile, our effect estimates were lower than a cross-sectional study among 50,593 participants from Fujian Province in China [OR = 0.81, 95%CI: 0.79, 0.83, per 0.1 unit] [22]. However, several previous studies have also reported insignificant associations of residential greenness with HBP or DM [16, 23]. A meta-analysis based on 6 studies also indicated that residential greenness is inversely but insignificantly associated with risks of diabetes [relative risk (RR) = 0.90, 95%CI: 0.79, 1.03] [18]. Based on the national representative CNSCKD survey with relatively large geographic and population scales, our study may provide robust empirical evidence supporting the beneficial effects of green spaces on HBP and DM.

An increasing number of studies have linked urbanization and SES to HBP and DM [4–8]. Regional urbanicity and individual SES profoundly shape the accessibility and utilization of environmental facilities, healthcare services, and residential greenness [29, 39, 40]. These factors may thus have interactive effects on HBP and DM, resulting in disparities in associations of residential greenness with HBP and DM across regions and individuals [30–32]. However, the modifying role of regional urbanicity and individual SES on associations of residential greenness with HBP and DM has remained unclear to date.

In this study, we observed weaker associations of NDVI with HBP and DM in rural regions in comparison to urban regions. Several studies have also yielded consistent findings. A cross-sectional study among 2,078 children residing in Munich and Wesel, Germany, indicated that the association between residential greenness and blood pressure is only significant among children residing in the urbanized Munich area [14]. A cohort study among 4.2 million adults in Switzerland also revealed more apparent protective effects of residential greenness on cardiovascular disease (CVD) mortality among individuals in urban regions [69]. A cohort study among 429,504 participants in Taiwan, China also found weaker protective effects of residential greenness on DM in less urbanized regions [20]. However, a cohort study among 25,639 older residents of East Anglia in the UK [19] and a cohort study among 6,814 participants in the US [21] yielded insignificant urban-rural differences. In addition, a study among 6,076 participants in the UK indicated more apparent associations between residential greenness and HBP in regions with higher area deprivation [51]. Several studies also found stronger associations in rural regions compared to urban regions [22, 28].

Considering the differences in urban-rural definitions across countries, this study further used the continuous DMSP/OLS NLI data to characterize regional urbanicity. Compared to the administrative urban-rural classification, NLI can more sensitively capture the geographically refined regional urbanicity level in real-world settings [46, 48, 49]. Our results indicated that along with the decrease in NLI levels, there were continuously decreasing inverse NDVI-DM associations, while no statistically significant effect modification by NLI on NDVI-HBP associations was observed. The results highlighted the unequal health effects of residential greenness on DM caused by urbanization.

Nevertheless, it should be noted that an increasing number of studies have highlighted the adverse health effects of light at night. Physiologically, exposure to light at night can disrupt the circadian rhythm by affecting the suprachiasmatic nucleus and further result in various disorders [70, 71]. Epidemiological studies have also linked light at night exposure to increased risks of cardiometabolic [72–75], mental [76, 77], and neoplastic disorders [78]. Accordingly, our results might characterize the dual roles of NLI as both an urbanicity indicator and a potential risk factor. Further studies are thus warranted to consider these complex interactions, in order to fully understand the unequal effects of greenness attributed to urbanization.

This study also found weaker NDVI-HBP and NDVI-DM associations among individuals with lower income and lower education levels compared to their counterparts. Similar findings have also been reported by a cohort study among 4.2 million adults in Switzerland [69]. Nevertheless, other studies have yielded different findings. Studies among 6,076 participants in the UK [51], 5,735 participants from two European cities [58], 9,354 children from 7 cities in China [79], and 24,845 urban dwellers from 33 communities in 3 cities in China [34] observed insignificant differences in greenness-HBP associations among individuals with different SES levels. Meanwhile, evidence from New South Wales, Australia [26], urban Bangladesh [35], the US [21], Ningbo City, China [36], rural regions in Henan Province, China [24], and 33 communities in 3 cities in China [50] reported no differences in greenness-DM associations when stratified by education level. The inconsistent findings may be attributable to differences in geographic location and population characteristics across different countries [18, 22, 28, 58]. Meanwhile, most previous studies were conducted based on relatively small geographic distribution and population scale [11, 33–38], which may be less capable of characterizing the socio-economic disparities in the general population.

To our knowledge, this is the first study to systematically evaluate the effect modification by regional urbanicity and individual SES on associations of residential greenness with HBP and DM. Our empirical study based on large population and geographic scales can provide robust and practical evidence for green space planning. However, several limitations of this study should be mentioned. First, the cross-sectional design may be subjected to causal confusion owing to information bias and the absence of temporal information. Second, the HBP and DM were characterized partly based on self-reported information, which may to some extent impact the accuracy of HBP and DM prevalence. Meanwhile, the single-time measurements may also overestimate HBP and DM prevalence [41]. Third, our study used residential greenness, but not actual time spent in green spaces, due to limited data accessibility. The exposure misclassification may also underestimate the association between green spaces and health outcomes. Fourth, the survey time is relatively early, which may not fully capture the modifying effects of regional urbanicity and individual SES in more recent years. Fifth, our results did not control the potential confounding effects of indoor temperature, which has been reported to be associated with HBP in previous studies [80, 81].

## 5. Conclusions

In summary, our national representative cross-sectional study revealed inverse associations of residential greenness with HBP and DM prevalence. Moreover, lower regional urbanicity and individual SES levels could attenuate the associations of residential greenness with HBP and DM. Our survey provides empirical evidence at large population and geographic scales supporting guided green space planning under the global context of urbanization, particularly for regions with less urbanicity and individuals with lower SES levels. Given the relatively insufficient evidence to date, further multi-center prospective studies are still warranted to confirm the associations, as well as the inequalities caused by urbanization and SES disparities.

## Abbreviations

BMI: Body Mass Index
CNSCKD: China National Survey of Chronic Kidney Disease
DALYs: Disability-Adjusted Life Years
DBP: Diastolic Blood Pressure
DM: Diabetes Mellitus
DMSP: Defense Meteorological Satellite Program
FPG: Fasting Plasma Glucose
GAM: Generalized Additive Model
GBD: Global Burden of Disease
HBP: High Blood Pressure
IQR: Interquartile Range
LMICs: Low- and Middle-Income Countries
NDVI: Normalized Difference Vegetation Index
NIR: Near-Infrared Radiation
NLI: Nighttime Light Index
OLS: Operational Line-scan System
OR: Odds Ratio
PPS: Probability-Proportional-to-Size
SBP: Systolic Blood Pressure
SES: Socio-Economic Status

## Supplementary information

**Additional file 1:** **Supplementary Method I.** The residential greenness, nighttime light index (NLI), ambient fine particulate matter (PM_2.5_), ozone (O_3_) pollution, and temperature remote sensing inversion datasets used in this study. **Table S1.** Baseline characteristics of the participants from the CNSCKD survey included in this study. **Table S2.** Spearman correlation coefficients of the NDVI and NLI in this study. **Table S3.** Detailed effect estimates for **Figure 2**. **Table S4.** Detailed effect estimates for **Figure 3**. **Table S5.** Detailed effect estimates for **Figure 4**. **Table S6.** Associations of residential NDVI level with prevalence of HBP and DM in the sensitivity analyses. **Figure S1.** Flowchart of the participants from the CNSCKD survey included in the final analyses. **Figure S2.** Exposure-response curves for associations of residential NDVI level with prevalence of HBP and DM at all the exposure metrics (Lag01∼Lag05) among the general population. **Figure S3.** Effect modification by NLI on associations of residential NDVI level with prevalence of HBP and DM at all the exposure metrics (Lag01∼Lag05).

## Declarations

### Availability of data and materials

The datasets used and/or analysed during the current study are available from the corresponding author on reasonable request.
