# Supplementary material for: Lower regional urbanicity and socioeconomic status attenuate associations of green spaces with hypertension and diabetes mellitus: a national representative cross-sectional study in China
Source: Environ Health Prev Med. 2024 Sep 7;29:47. doi: 10.1265/ehpm.24-00121 (PMC11391273; doi:10.1265/ehpm.24-00121)
Supplement: Supplementary file 1 — Additional file 1: Supplementary Method I. The residential greenness, nighttime light index (NLI), ambient fine particulate matter (PM2.5), ozone (O3) pollution, and temperature remote sensing inversion datasets used in this study. Table S1. Baseline characteristics of the participants from the CNSCKD survey included in this study. Table S2. Spearman correlation coefficients of the NDVI and NLI in this study. Table S3. Detailed effect estimates for Figure 2. Table S4. Detailed effect estimates for Figure 3. Table S5. Detailed effect estimates for Figure 4. Table S6. Associations of residential NDVI level with prevalence of HBP and DM in the sensitivity analyses. Figure S1. Flowchart of the participants from the CNSCKD survey included in the final analyses. Figure S2. Exposure-response curves for associations of residential NDVI level with prevalence of HBP and DM at all the exposure metrics (Lag01∼Lag05) among the general population. Figure S3. Effect modification by NLI on associations of residential NDVI level with prevalence of HBP and DM at all the exposure metrics (Lag01∼Lag05). [file ehpm-29-047-s001.docx]

**Lower regional urbanicity and socioeconomic status attenuate associations of green spaces with hypertension and diabetes mellitus: A national representative cross-sectional study in China**

**Supplementary Method I.** The residential greenness, nighttime light index (NLI), ambient fine particulate matter (PM_2.5_), ozone (O_3_) pollution, and temperature remote sensing inversion datasets used in this study.

**Table S1.** Baseline characteristics of the participants from the CNSCKD survey included in this study.

**Table S2.** Spearman correlation coefficients of the NDVI and NLI in this study.

**Table S3.** Detailed effect estimates for **Figure 2**.

**Table S4.** Detailed effect estimates for **Figure 3**.

**Table S5.** Detailed effect estimates for **Figure 4**.

**Table S6.** Associations of residential NDVI level with prevalence of HBP and DM in the sensitivity analyses.

**Figure S1.** Flowchart of the participants from the CNSCKD survey included in the final analyses.

**Figure S2.** Exposure-response curves for associations of residential NDVI level with prevalence of HBP and DM at all the exposure metrics (Lag01~Lag05) among the general population.

**Figure S3.** Effect modification by NLI on associations of residential NDVI level with prevalence of HBP and DM at all the exposure metrics (Lag01~Lag05).

**Supplementary Method I.** The residential greenness, nighttime light index (NLI), ambient fine particulate matter (PM_2.5_), ozone (O_3_) pollution, and temperature remote sensing inversion datasets used in this study.

**(1) Residential greenness, normalized difference vegetation index (NDVI)**

Considering that plants have a high reflectance in the near-infrared radiation (NIR) channel and a low reflectance in the red reflectance (Red) channel, the NDVI is a normalized transform of the NIR to Red ratio calculated using the formula:

$$NDVI= \frac{NIR-Red}{NIR+Red}$$

The NDVI has the advantage of minimizing types of band-correlated noise and influences attributed to variations in direct/diffuse irradiance, clouds and cloud shadows, sun and view angles, topography, and atmospheric attenuation.

In this study, we acquired SPOT VEGETATION NDVI data from the year 2002 to 2010 with a spatial resolution of 1 km*1 km and a temporal resolution of 10 days in China from the Resource and Environment Data Cloud Platform (<http://www.resdc.cn/>) (Sun et al. 2020). All the NDVI datasets underwent geometric and atmospheric corrections, and maximum value composite (MVC) was used to further remove cloud, atmosphere, and sun altitude angle influence (Deronde et al. 2014; Guo et al. 2014).

**(2) Regional urbanicity, nighttime light index (NLI)**

The NLI used in this study were obtained from the Defense Meteorological Satellite Program (DMSP)/Operational Line-scan System (OLS) nighttime light v4 stable average visible data from the National Oceanic and Atmospheric Administration National Geophysical Data Center (<http://ngdc.noaa.gov/eog/dmsp/downloadV4composites.html>). The ridgeline sampling regression method was further used to calibrate the DMSP/OLS data to generate a consistent NLI time series (Zhang et al. 2016). The NLI data can approximate regional gross domestic product (GDP) and socio-economic status and thus have been widely used to characterize regional urbanicity levels (Liang et al. 2021; Ma et al. 2012; Wang et al. 2021). To characterize the regional urbanicity level, we generated buffer zones with a 10 km radius and calculated the average DMSP/OLS NLI grid value in each buffer zone. More detailed methods on the DMSP/OLS NLI have been reported in our previous work (Liang et al. 2021).

**(3) Ambient air pollution and temperature**

Due to the lack of a regulatory monitoring platform before the year 2012 in China, administrative ambient air pollution and meteorological monitoring data during the study period (2007–2010) were not accessible. Therefore, we assigned the exposure data for the participants using acknowledged ground-level satellite remote sensing inversion datasets.

The ambient PM_2.5_ dataset was generated based on a multi-source combination of aerosol optical depth (AOD) retrievals from the NASA Moderate Resolution Imaging Spectroradiometer (MODIS), Multiangle Imaging Spectroradiometer (MISR), and Sea-Viewing Wide Field-of-View Sensor (SeaWiFS). Based on AOD data, near-surface PM_2.5_ concentrations were obtained using the GEOS-Chem chemical transport model and the geographically weighted regression (GWS) model. (van Donkelaar et al. 2016).

The ambient O_3_ daily maximum 8h average dataset was constructed through a high-performance random forest model based on available meteorological variables, satellite data, chemical transport model output, geographical variables, and socio-economic variables (Ma et al. 2022; Wang et al. 2022).

This study accessed the temperature data through the Princeton re-analysis, Global Land Data Assimilation System, Global Energy and Water Cycle Experiment-Surface Radiation Budget radiation, and Tropical Rainfall Measuring Mission precipitation data (He et al. 2020; Kun and Jie 2018; Liang et al. 2021; Yang et al. 2010). The ambient temperature data were generated based on the China Meteorological Forcing Dataset (CMFD) (He et al. 2020; Kun and Jie 2018; Yang et al. 2010).

Studies reported that the datasets were highly consistent with out-of-sample cross-validated monitoring data in China and other areas (Geddes et al. 2016; Ma et al. 2022), and have been widely used in nationwide epidemiological studies (Cooper et al. 2022; Liang et al. 2021; Ma et al. 2022; Wang et al. 2022; Yang et al. 2022).

**Table S1.** Baseline characteristics of the participants from the CNSCKD survey included in this study.

| **Characteristics** | **HBP & Non-HBP**  **(N =** **44629)** | **DM & non-DM**  **(N =** **44833)** |
| --- | --- | --- |
| Region |  |  |
| Rural | 21596 (48.4%) | 21714 (48.4%) |
| Urban | 23033 (51.6%) | 23119 (51.6%) |
| Income (*yuan*, per capita month) |  |  |
| ≤ 500 | 15925 (35.7%) | 15972 (35.6%) |
| 500~1000 | 11187 (25.1%) | 11227 (25.0%) |
| > 1000 | 9171 (20.5%) | 9194 (20.5%) |
| Not reported | 8346 (18.7%) | 8440 (18.8%) |
| Education |  |  |
| Below primary school | 4619 (10.3%) | 4620 (10.3%) |
| Primary school | 8354 (18.7%) | 8372 (18.7%) |
| Middle school | 12421 (27.8%) | 12453 (27.8%) |
| High school | 11146 (25.0%) | 11196 (25.0%) |
| College and upper | 7977 (17.9%) | 8073 (18.0%) |
| Not reported | 112 (0.3%) | 119 (0.3%) |
| Sex |  |  |
| Male | 19130 (42.9%) | 19220 (42.9%) |
| Female | 25499 (57.1%) | 25613 (57.1%) |
| Age (years) | 49.6±15.2 | 49.5±15.3 |
| BMI (kg/m^2^) | 23.8±3.7 | 23.8±3.7 |
| Smoking status |  |  |
| Non-smoking | 34137 (76.5%) | 34293 (76.5%) |
| Current smoking | 10457 (23.4%) | 10502 (23.4%) |
| Not reported | 35 (0.1%) | 38 (0.1%) |
| Alcohol drinking |  |  |
| < 1 time a week | 37658 (84.4%) | 37830 (84.4%) |
| 1~2 times a week | 2427 (5.4%) | 2441 (5.4%) |
| > 3 times a week | 4453 (10.0%) | 4468 (10.0%) |
| Not reported | 91 (0.2%) | 94 (0.2%) |
| Physical activity level |  |  |
| < 3 hours/week | 24806 (55.6%) | 24879 (55.5%) |
| ≥ 3 hours/week | 10645 (23.9%) | 10683 (23.8%) |
| Not reported | 9178 (20.6%) | 9271 (20.7%) |
| Dietary vegetable intake |  |  |
| ≥ 500 g/day | 16336 (36.6%) | 16422 (36.6%) |
| 250–500 g/day | 19264 (43.2%) | 19341 (43.1%) |
| < 250 g/day | 6376 (14.3%) | 6408 (14.3%) |
| Not reported | 2653 (5.9%) | 2662 (5.9%) |

Note: Distributions are showed as number (percentage) for categorical variables or Mean ± Standard Deviation (SD) for continuous variables.

**Table S2.** Spearman correlation coefficients of the NDVI and NLI in this study.

| **Indicator** | **NDVILag01** | **NDVILag02** | **NDVILag03** | **NDVILag04** | **NDVILag05** | **NLILag01** | **NLILag02** | **NLILag03** | **NLILag04** | **NLILag05** |
| --- | --- | --- | --- | --- | --- | --- | --- | --- | --- | --- |
| **NDVILag01** | — |  |  |  |  |  |  |  |  |  |
| **NDVILag02** | 0.997* | — |  |  |  |  |  |  |  |  |
| **NDVILag03** | 0.995* | 0.996* | — |  |  |  |  |  |  |  |
| **NDVILag04** | 0.991* | 0.993* | 0.997* | — |  |  |  |  |  |  |
| **NDVILag05** | 0.986* | 0.990* | 0.994* | 0.997* | — |  |  |  |  |  |
| **NLILag01** | -0.756* | -0.749* | -0.762* | -0.743* | -0.734* | — |  |  |  |  |
| **NLILag02** | -0.762* | -0.754* | -0.769* | -0.750* | -0.740* | 0.995* | — |  |  |  |
| **NLILag03** | -0.766* | -0.759* | -0.773* | -0.754* | -0.744* | 0.993* | 0.998* | — |  |  |
| **NLILag04** | -0.776* | -0.768* | -0.781* | -0.762* | -0.752* | 0.990* | 0.995* | 0.998* | — |  |
| **NLILag05** | -0.773* | -0.766* | -0.779* | -0.760* | -0.750* | 0.989* | 0.994* | 0.998* | 0.999* | — |

***** *P*<0.05.

**Table S3.** Detailed effect estimates for **Figure 2**.

| **Disease** | **Main Model** |
| --- | --- |
| **Hypertension** |  |
| Lag01 | 0.87 (0.81, 0.93) |
| Lag02 | 0.86 (0.81, 0.93) |
| Lag03 | 0.86 (0.81, 0.93) |
| Lag04 | 0.88 (0.82, 0.94) |
| Lag05 | 0.87 (0.82, 0.93) |
| **Diabetes mellitus** |  |
| Lag01 | 0.83 (0.77, 0.89) |
| Lag02 | 0.82 (0.76, 0.88) |
| Lag03 | 0.79 (0.74, 0.85) |
| Lag04 | 0.79 (0.73, 0.84) |
| Lag05 | 0.79 (0.74, 0.85) |

**Note:** Results were shown as estimated odds ratio (OR) and its 95% confidence interval (95% CI) of HBP or DM prevalence per interquartile range (0.26) increase in NDVI value. Covariates included administrative region (urban/rural), income, education, DMSP/OLS NLI, sex, age, body mass index (BMI), smoking status, alcohol drinking, physical activity level, dietary vegetable intake, and season.

**Table S4.** Detailed effect estimates for **Figure 3**.

| **Disease** | **Rural** | **Urban** | **P-value** |
| --- | --- | --- | --- |
| **Hypertension** |  |  |  |
| Lag01 | 1.00 (0.91, 1.11) | 0.89 (0.81, 0.97) | 0.077 |
| Lag02 | 1.03 (0.93, 1.14) | 0.86 (0.79, 0.94) | 0.011 |
| Lag03 | 1.03 (0.93, 1.13) | 0.86 (0.79, 0.94) | 0.011 |
| Lag04 | 1.05 (0.95, 1.16) | 0.87 (0.80, 0.94) | 0.005 |
| Lag05 | 1.04 (0.94, 1.15) | 0.85 (0.79, 0.93) | 0.003 |
| **Diabetes mellitus** |  |  |  |
| Lag01 | 1.11 (1.00, 1.23) | 0.57 (0.52, 0.63) | <0.001 |
| Lag02 | 1.15 (1.03, 1.28) | 0.55 (0.50, 0.60) | <0.001 |
| Lag03 | 1.09 (0.98, 1.21) | 0.54 (0.50, 0.59) | <0.001 |
| Lag04 | 1.11 (1.00, 1.23) | 0.55 (0.50, 0.60) | <0.001 |
| Lag05 | 1.14 (1.03, 1.26) | 0.55 (0.50, 0.60) | <0.001 |

Note: Results were shown as estimated odds ratio (OR) and its 95% confidence interval (95% CI) of HBP or DM prevalence per interquartile range (0.26) increase in NDVI value. Covariates included income, education, sex, age, body mass index (BMI), smoking status, alcohol drinking, physical activity level, dietary vegetable intake, and season. The subgroup differences were evaluated using the Z-test statistics.

**Table S5.** Detailed effect estimates for **Figure 4**.

| **Disease** | **Lower group** | **Higher group** | **P-value** |
| --- | --- | --- | --- |
| **Income** | | | |
| **Hypertension** |  |  |  |
| Lag01 | 0.99 (0.91, 1.08) | 0.56 (0.46, 0.68) | <0.001 |
| Lag02 | 1.00 (0.92, 1.09) | 0.52 (0.43, 0.64) | <0.001 |
| Lag03 | 1.00 (0.92, 1.09) | 0.52 (0.43, 0.64) | <0.001 |
| Lag04 | 1.01 (0.93, 1.10) | 0.55 (0.46, 0.66) | <0.001 |
| Lag05 | 1.01 (0.93, 1.09) | 0.54 (0.45, 0.64) | <0.001 |
| **Diabetes mellitus** |  |  |  |
| Lag01 | 1.13 (1.04, 1.24) | 0.68 (0.55, 0.84) | <0.001 |
| Lag02 | 1.15 (1.05, 1.26) | 0.61 (0.50, 0.75) | <0.001 |
| Lag03 | 1.09 (1.00, 1.19) | 0.58 (0.48, 0.71) | <0.001 |
| Lag04 | 1.08 (0.99, 1.18) | 0.57 (0.47, 0.69) | <0.001 |
| Lag05 | 1.08 (0.99, 1.18) | 0.57 (0.47, 0.69) | <0.001 |
| **Education** | | | |
| **Hypertension** |  |  |  |
| Lag01 | 0.95 (0.87, 1.04) | 0.69 (0.61, 0.77) | <0.001 |
| Lag02 | 0.96 (0.87, 1.05) | 0.67 (0.59, 0.75) | <0.001 |
| Lag03 | 0.95 (0.87, 1.04) | 0.67 (0.59, 0.76) | <0.001 |
| Lag04 | 0.96 (0.88, 1.05) | 0.69 (0.61, 0.77) | <0.001 |
| Lag05 | 0.95 (0.87, 1.04) | 0.68 (0.61, 0.77) | <0.001 |
| **Diabetes mellitus** |  |  |  |
| Lag01 | 1.01 (0.92, 1.10) | 0.62 (0.55, 0.71) | <0.001 |
| Lag02 | 1.02 (0.93, 1.12) | 0.59 (0.52, 0.68) | <0.001 |
| Lag03 | 0.98 (0.89, 1.07) | 0.58 (0.51, 0.66) | <0.001 |
| Lag04 | 0.97 (0.89, 1.06) | 0.58 (0.51, 0.65) | <0.001 |
| Lag05 | 0.98 (0.89, 1.07) | 0.58 (0.51, 0.66) | <0.001 |

Note: Results were shown as estimated odds ratio (OR) and its 95% confidence interval (95% CI) of HBP or DM prevalence per interquartile range (0.26) increase in NDVI value. Covariates included administrative region (urban/rural), income, education, DMSP/OLS NLI, sex, age, body mass index (BMI), smoking status, alcohol drinking, physical activity level, dietary vegetable intake, and season. The income/education was not included in their separate model. The subgroup differences were evaluated using the Z-test statistics.

**Table S6.** Associations of residential NDVI level with prevalence of HBP and DM in the sensitivity analyses.

| **Pollutant** | **Main Model^a^** | **PM_2.5_ adjusted^b^** | **O_3_ adjusted^c^** | **Temperature adjusted^d^** |
| --- | --- | --- | --- | --- |
| **Hypertension** |  |  |  |  |
| Lag01 | 0.87 (0.81, 0.93) | 0.84 (0.78, 0.90) | 0.86 (0.80, 0.93) | 0.86 (0.80, 0.92) |
| Lag02 | 0.86 (0.81, 0.93) | 0.84 (0.78, 0.90) | — | 0.85 (0.79, 0.92) |
| Lag03 | 0.86 (0.81, 0.93) | 0.85 (0.79, 0.91) | — | 0.85 (0.79, 0.92) |
| Lag04 | 0.88 (0.82, 0.94) | 0.86 (0.80, 0.93) | — | 0.87 (0.81, 0.93) |
| Lag05 | 0.87 (0.82, 0.93) | 0.86 (0.80, 0.92) | — | 0.86 (0.80, 0.92) |
| **Diabetes mellitus** |  |  |  |  |
| Lag01 | 0.83 (0.77, 0.89) | 0.82 (0.76, 0.89) | 0.83 (0.77, 0.89) | 0.79 (0.74, 0.86) |
| Lag02 | 0.82 (0.76, 0.88) | 0.82 (0.76, 0.88) | — | 0.78 (0.73, 0.84) |
| Lag03 | 0.79 (0.74, 0.85) | 0.79 (0.73, 0.85) | — | 0.76 (0.70, 0.82) |
| Lag04 | 0.79 (0.73, 0.84) | 0.78 (0.73, 0.84) | — | 0.76 (0.71, 0.82) |
| Lag05 | 0.79 (0.74, 0.85) | 0.79 (0.73, 0.85) | — | 0.77 (0.71, 0.82) |

Note: Results were shown as estimated odds ratio (OR) and its 95% confidence interval (95% CI) of HBP or DM prevalence per interquartile range (0.26) increase in NDVI value.

^a^ Covariates included administrative region (urban/rural), income, education, DMSP/OLS NLI, sex, age, body mass index (BMI), smoking status, alcohol drinking, physical activity level, dietary vegetable intake, and season.

^b^ Models additionally adjusted for PM_2.5_.

^c^ Models additionally adjusted for O_3_. Analyses additionally adjusted for O_3_ at Lag02~Lag05 were not conducted due to the limited data availability on O_3_-8h maximum concentration before the year 2005.

^d^ Models additionally adjusted for temperature.

**
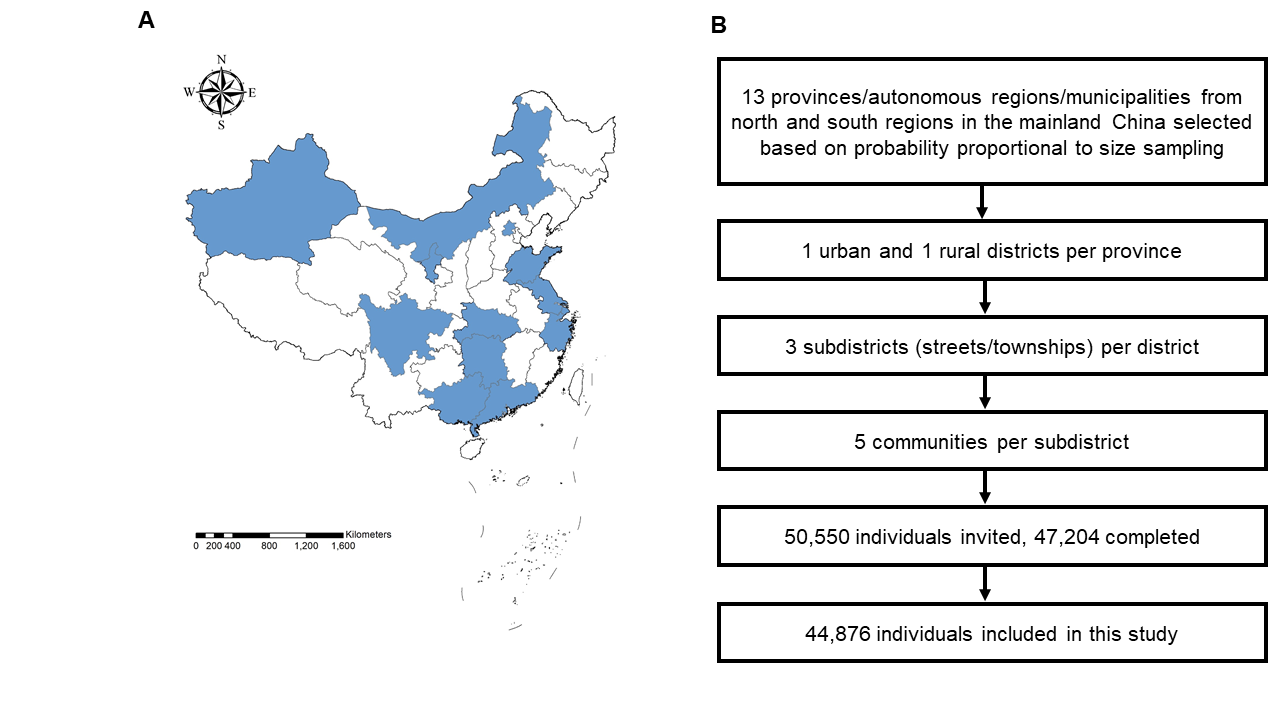
**

**Figure S1.** Flowchart of the participants from the CNSCKD survey included in the final analyses. (A) Study location; (B) Sampling procedures. Note: The included provinces/autonomous regions/municipalities were Beijing, Guangdong, Guangxi Zhuang Autonomous Region, Hubei, Hunan, Inner Mongolia Autonomous Region, Jiangsu, Ningxia Hui Autonomous Region, Shanghai, Shandong, Sichuan, Xinjiang Uyghur Autonomous Region, and Zhejiang.


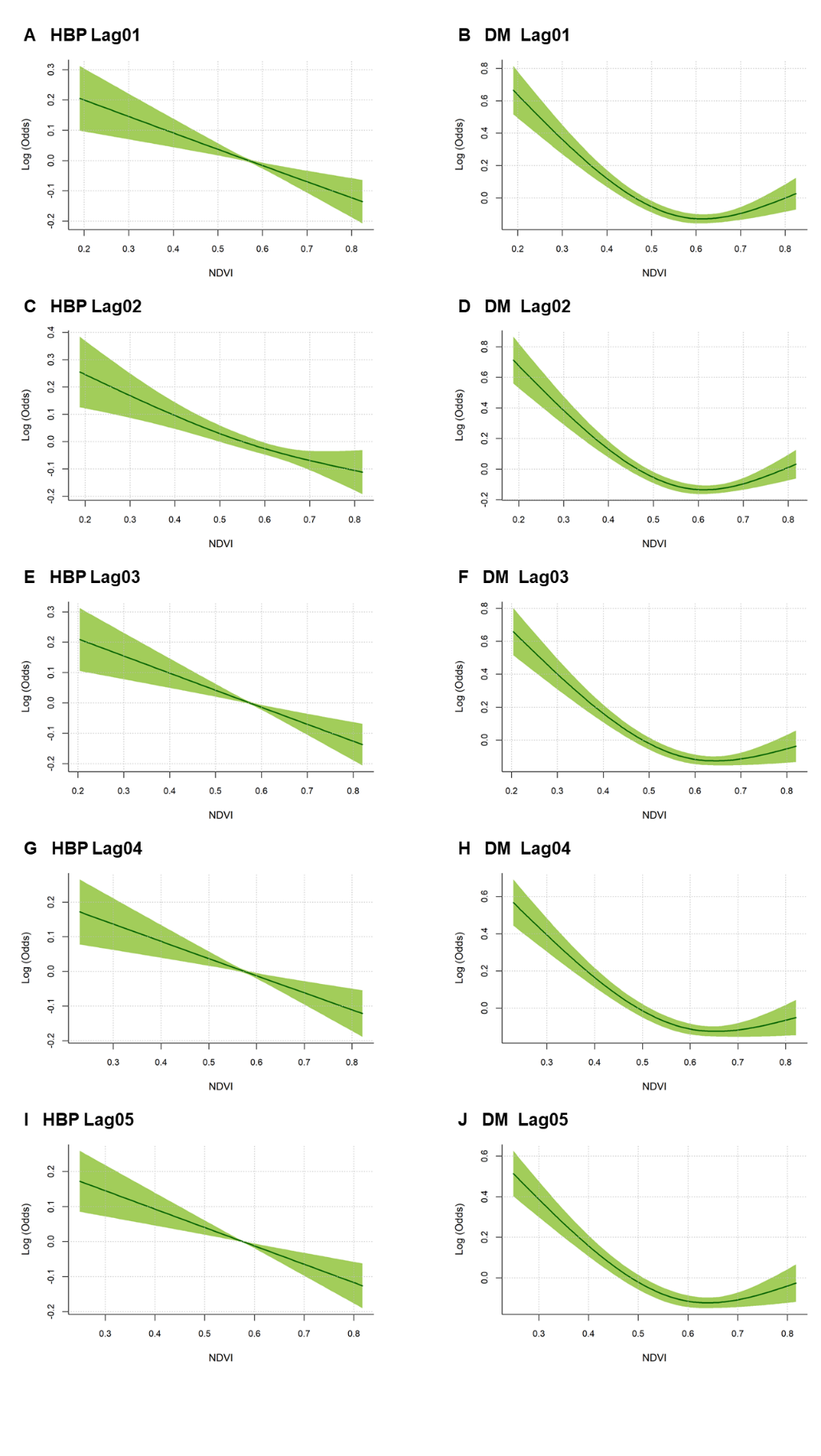


**Figure S2.** Exposure-response curves for associations of residential NDVI level with prevalence of HBP and DM at all the exposure metrics (Lag01~Lag05) among the general population. Note: Covariates included administrative region (urban/rural), income, education, DMSP/OLS NLI, sex, age, body mass index (BMI), smoking status, alcohol drinking, physical activity level, dietary vegetable intake, and season.


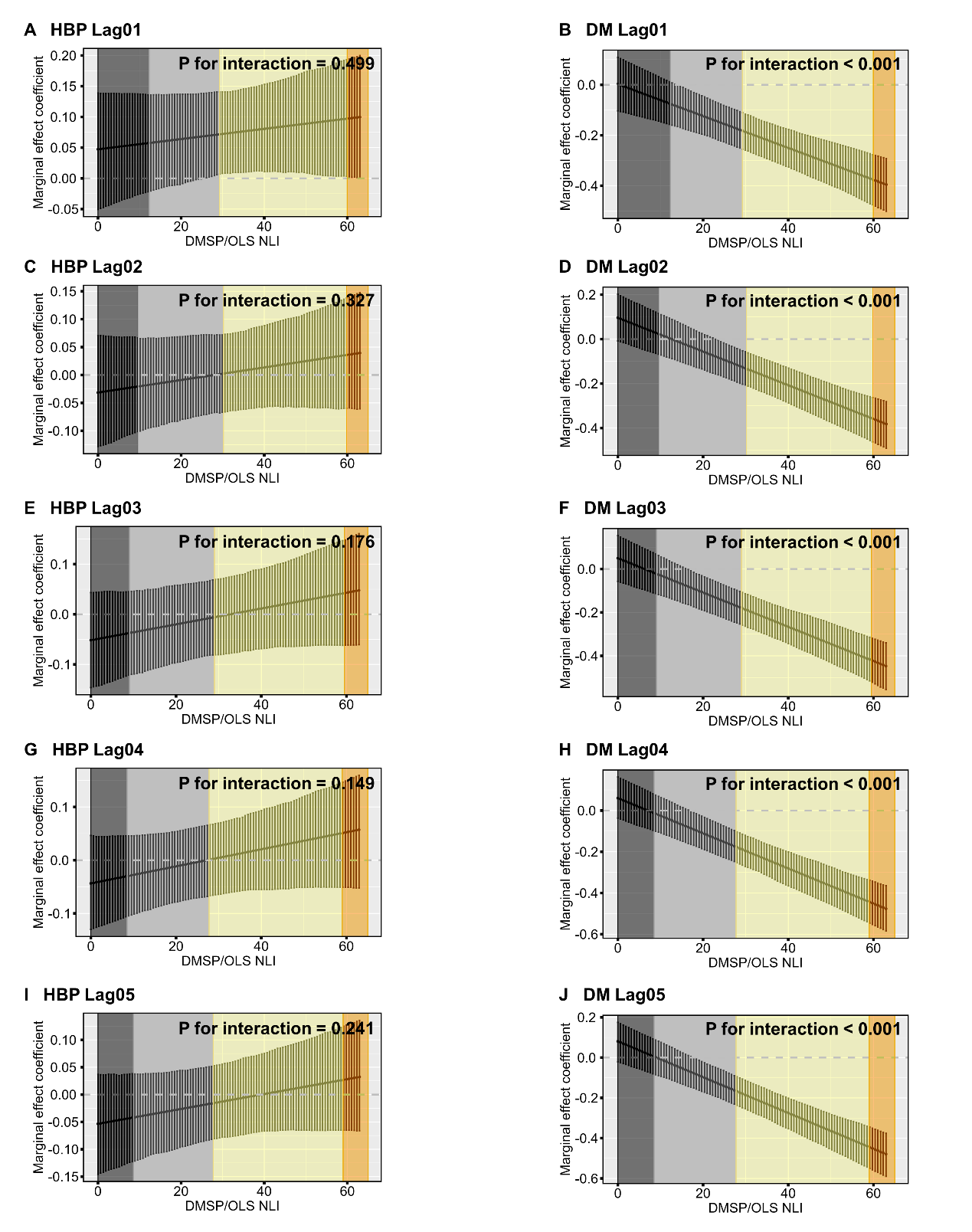


**Figure S3.** Effect modification by NLI on associations of residential NDVI level with prevalence of HBP and DM at all the exposure metrics (Lag01~Lag05). Note: Results were shown as estimated conditional effect and its 95% confidence interval (95% CI). Covariates included income, education, sex, age, body mass index (BMI), smoking status, alcohol drinking, physical activity level, dietary vegetable intake, and season. P for interaction indicated the statistical significance for the multiplicative interaction terms of NDVI and DMSP/OLS NLI.

**References:**

Cooper MJ, Martin RV, Hammer MS, Levelt PF, Veefkind P, Lamsal LN, et al. 2022. Global fine-scale changes in ambient no(2) during covid-19 lockdowns. Nature 601:380-387.

Deronde B, Debruyn W, Gontier E, Goor E, Jacobs T, Verbeiren S, et al. 2014. 15 years of processing and dissemination of spot-vegetation products. International Journal of Remote Sensing 35:2402-2420.

Geddes JA, Martin RV, Boys BL, van Donkelaar A. 2016. Long-term trends worldwide in ambient no2 concentrations inferred from satellite observations. Environ Health Perspect 124:281-289.

Guo B, Zhou Y, Wang SX, Tao HP. 2014. The relationship between normalized difference vegetation index (ndvi) and climate factors in the semiarid region: A case study in yalu tsangpo river basin of qinghai-tibet plateau. Journal of Mountain Science 11:926-940.

He J, Yang K, Tang W, Lu H, Qin J, Chen Y, et al. 2020. The first high-resolution meteorological forcing dataset for land process studies over china. Scientific Data 7:25.

Kun Y, Jie HE. 2018. China meteorological forcing dataset (1979-2018). National Tibetan Plateau Data Center.

Liang Z, Wang W, Wang Y, Ma L, Liang C, Li P, et al. 2021. Urbanization, ambient air pollution, and prevalence of chronic kidney disease: A nationwide cross-sectional study. Environ Int 156:106752.

Ma R, Ban J, Wang Q, Zhang Y, Yang Y, Li S, et al. 2022. Full-coverage 1km daily ambient pm2.5 and o3 concentrations of china in 2005–2017 based on a multi-variable random forest model. Earth Syst Sci Data 14:943-954.

Ma T, Zhou C, Tao P, Haynie S, Fan J. 2012. Quantitative estimation of urbanization dynamics using time series of dmsp/ols nighttime light data: A comparative case study from china's cities. Remote Sensing of Environment 124.

Sun G, Guo B, Zang W, Huang X, Han B, Yang X, et al. 2020. Spatial–temporal change patterns of vegetation coverage in china and its driving mechanisms over the past 20 years based on the concept of geographic division. Geomatics, Natural Hazards and Risk 11:2263-2281.

van Donkelaar A, Martin RV, Brauer M, Hsu NC, Kahn RA, Levy RC, et al. 2016. Global estimates of fine particulate matter using a combined geophysical-statistical method with information from satellites, models, and monitors. Environ Sci Technol 50:3762-3772.

Wang Q, Zhang Y, Ban J, Zhu H, Xu H, Li T. 2021. The relationship between population heat vulnerability and urbanization levels: A county-level modeling study across china. Environ Int 156:106742.

Wang Q, Zhu H, Xu H, Lu K, Ban J, Ma R, et al. 2022. The spatiotemporal trends of pm2.5- and o3-related disease burden coincident with the reduction in air pollution in china between 2005 and 2017. Resources, Conservation and Recycling 176:105918.

Yang C, Wang W, Wang Y, Liang Z, Zhang F, Chen R, et al. 2022. Ambient ozone pollution and prevalence of chronic kidney disease: A nationwide study based on the china national survey of chronic kidney disease. Chemosphere 306:135603.

Yang K, He J, Tang W, Qin J, Cheng CCK. 2010. On downward shortwave and longwave radiations over high altitude regions: Observation and modeling in the tibetan plateau. Agricultural and Forest Meteorology 150:38-46.

Zhang Q, Pandey B, Seto KC. 2016. A robust method to generate a consistent time series from dmsp/ols nighttime light data. IEEE Transactions on Geoscience & Remote Sensing 54:5821-5831.
